# Autologous skin graft intersphincteric implantation in anal fistula treatment (ASGIIFT) – A novel surgical technique in the treatment of complex transsphincteric anal fistulas

**DOI:** 10.1111/codi.70407

**Published:** 2026-02-16

**Authors:** Damir Karlović, Dorian Kršul, Dora Fučkar Čupić, Marko Zelić

**Affiliations:** ^1^ Department of Digestive Surgery University Hospital Rijeka Rijeka Croatia; ^2^ Center for Digestive and Metabolic Medicine, Faculty of Medicine University of Rijeka Rijeka Croatia; ^3^ Department of Pathology, Faculty of Medicine University of Rijeka Rijeka Croatia

**Keywords:** anal fistula, autograft, faecal incontinence, implantation

## Abstract

**Aim:**

This study aimed to evaluate whether implantation of an autologous skin graft in the intersphincteric space, as part of the ASGIIFT procedure, improves the primary healing of complex transsphincteric cryptoglandular anal fistulas.

**Methods:**

A prospective observational IDEAL stage 2a study was conducted at a tertiary referral centre for minimally invasive colorectal surgery and proctology in Croatia between September 2021 and January 2023, with an 18‐month follow‐up. Preoperative pelvic MRI was performed in all cases, and 40 adult patients who met the inclusion criteria were included in the study. The primary outcome was the postoperative primary healing rate which was defined clinically. Secondary outcomes included postoperative continence disturbance, postoperative pain, time of healing and other postoperative complications (Wexner score and VAS – Visual Analogue Scale were used). The ASGIIFT procedure includes all standard steps of the LIFT technique (ligation of the intersphincteric fistula tract), with the addition of a pre‐prepared autologous dermal graft placed into the intersphincteric space. The study was approved by the institutional ethics committee.

**Results:**

Primary clinical healing was achieved in 35 patients (87.5%) within a median of 4 weeks postoperatively (range 3–6 weeks). Five initially unhealed patients showed conversion from transsphincteric to intersphincteric fistula during the follow‐up period and were subsequently treated by fistulotomy without complications. No patient experienced worsening continence, and no serious postoperative complications occurred.

**Conclusion:**

ASGIIFT appears to be a safe and feasible technique for treating transsphincteric anal fistulas, showing promising early results in this single‐centre IDEAL 2a study. Further prospective comparative studies are warranted to validate these initial findings.

**What does this paper add to the literature?**

This IDEAL stage 2a evaluation of the ASGIIFT (Autologous Skin Graft Intersphincteric Implantation in Anal Fistula Treatment) novel sphincter preserving technique adds early evidence of its feasibility and safety for managing complex high transsphincteric anal fistulas, with encouraging initial healing and low complication rates.

## INTRODUCTION

The treatment of cryptoglandular complex anal fistulas presents a significant challenge for surgeons. The fistula tract consist of a tract lined with granulation tissue, wich supports chronic inflammation and prevents spontaneous healing [1].

Principles in the treatment of anal fistulas to achieve high healing rates include: resolving the internal fistula opening, addressing the intersphincteric space, adequately identifying and treating the primary and any secondary fistula tracts, removing all granulation tissue within the tract, ensuring continuous postoperative drainage and preserving the integrity of the sphincter complex [2, 3, 4, 5].

Although numerous surgical techniques have been proposed, a universally accepted ‘gold standard’ treatment is still lacking due to the complexity and variability of anal fistulas [6, 7, 8, 9, 10, 11, 12, 13].

The concept of intersphincteric space involvement and the anal gland theory as the underlying trigger for anal fistula development has persisted throughout history. Surgeons have attempted to address this challenge by developing different surgical strategies [14, 15].

Matos et al. also emphasised the importance of the intersphincteric space. They introduced a sphincter‐preserving approach that included several critical steps: eradication of cryptoglandular sepsis via intersphincteric access, closure of the internal opening, complete excision of the fistula complex, primary repair of the tract across the external sphincter and primary wound closure under antibiotic coverage [16].

This approach later influenced the development of Rojanasakul's LIFT technique (ligation of intersphincteric fistula tract) [7].

To improve outcome of the LIFT technique, implantation of a bioprosthetic graft in the intersphincteric space during LIFT procedure has been described by Ellis—BioLIFT technique [17].

Based on the previously mentioned evidence, we developed the ASGIIFT procedure. This technique was designed to address the challenge of managing the intersphincteric space while avoiding the use of expensive prosthetic materials by utilising autologous dermal tissue – an accessible and cost‐effective alternative. The underlying principle is that a pre‐prepared dermal graft, implanted into the intersphincteric space, promotes neoangiogenesis and healing while stabilising the intersphincteric space after treatment, facilitating fistula resolution and reducing the risk of recurrence. The average size of the autologous graft was determined by considering the typical dimensions of bioprosthetic material used in the BioLIFT procedure, as well as in combined LIFT and fistula plug techniques. Prior to this study, we conducted a small (unpublished) pilot study on five patients, approved by the hospital ethics committee and with informed consent obtained from all participants. All five patients achieved complete healing, providing preliminary evidence of feasibility and safety and supporting the rationale to proceed with the current IDEAL stage 2a evaluation. This pilot phase represents the iterative modification and internal refinement of the ASGIIFT technique, while all other procedural steps remain consistent with the standard LIFT technique except for the autologous graft implantation.

Similar principles of dermal grafting have long been employed successfully in plastic and reconstructive surgery as regenerative material [18].

Regarding the learning curve, the ASGIIFT procedure is technically straightforward for an experienced colorectal surgeon with prior proficiency in LIFT procedures. In our series, no additional major training was required beyond standard LIFT experience, and operating times remained consistent across cases, indicating reproducibility of the technique within the skilled surgical team.

According to the IDEAL framework for surgical innovation, this study represents a stage 2a evaluation, focusing on feasibility and safety in a limited patient series. The ASGIIFT procedure was developed and refined internally by the study team in accordance with this framework, ensuring a structured evaluation of the novel technique while maintaining patient safety and methodological consistency. Although no formal external steering group was established, all procedural steps were carefully planned, reviewed and standardised within the team prior to implementation.

## METHOD

### Study design and patient selection

A prospective observational study (IDEAL stage 2a) was conducted at the Department of Digestive Surgery, University Hospital Rijeka, a tertiary referral centre for minimally invasive colorectal surgery and proctology in Croatia, between September 2021 and January 2023, with an 18‐month follow‐up. All patients provided written informed consent for the procedure, and the principles of the Declaration of Helsinki were followed. The study was approved by the hospital's Institutional Ethics Commettee (Approval No: 2170‐29‐02/1‐25‐2).

Patients had clinical examination in the office, which included digital rectal examination and examination with a linear metal probe. All patients were referred for magnetic resonance imaging (MRI) of the pelvis for preoperative planning. Following preoperative assessment, patients were scheduled for the ASGIIFT procedure based on MRI findings and the inclusion criteria (40 adult patients).

Patients eligible for inclusion had complex cryptoglandular transsphincteric anal fistulas involving more than 30% of the external anal sphincter, with a single tract, a maximum length of 10 cm on preoperative MRI, and no significant extrasphincteric abscesses (≤1 cm in diameter). Of the 40 anal fistulas, 28 were posteriorly located and 12 were anterior (lithotomy position). Transsphincteric fistulas with intersphincteric or extrasphincteric collections and fistulas extending into the ischioanal fossa were excluded. Patients with supra‐ or extrasphincteric fistulas, low simple transsphincteric fistulas or Crohn's disease were also excluded from the study.

Preoperative seton placement was not routinely performed; only 12 patients had setons in place from previous surgical procedures prior to ASGIIFT treatment.

All procedures were performed by experienced coloproctologists, each with a minimum of 5 years' experience in treating complex anal fistulas using different sphincter‐preserving techniques and performing at least 80 such operations annually.

Follow‐up was conducted in an outpatient setting, where gentle debridement of the fistula tract under local anaesthesia and saline irrigation of the wound were performed. The first follow‐up visit was scheduled for the third or fourth postoperative day, followed by weekly visits as usual in our practice after anal fistula treatment with any sphincter preserving technique. After complete healing, patients were monitored every 1–2 months, or earlier if necessary.

Healing was defined as complete epithelialisation of the skin at the site of the previous external fistula opening, with no pain, swelling or discharge in the perianal region, or other signs of recurrence during the follow‐up period. Postoperative assessment of healing was based on structured clinical follow‐up by experienced coloproctologists. Routine postoperative imaging (MRI or ultrasound) was not performed, as it is not standard practice in our unit. While imaging can be useful for research purposes, it was not feasible for all patients due to logistical and cost considerations. Instead, clinical evaluation using stringent criteria was employed, which provides a robust and reliable measure of fistula healing. To ensure comprehensive assessment and timely detection of delayed recurrences, all patients were followed for up to 18 months postoperatively, with weekly visits until complete epithelialisation and subsequent follow‐up every 1–2 months.

### Operative technique

#### Preparing the patient for surgery

All patients underwent bowel preparation prior to surgery. Preoperative antibiotic prophylaxis was administered intravenously 30–60 min before the procedure, using ciprofloxacin 400 mg (or gentamicin 160 mg in cases of allergy) and metronidazole 500 mg. Patients were also advised to stop smoking and avoid alcohol.

#### Procedure

Patients were placed in the lithotomy position. All procedures were performed under either spinal or general anaesthesia (34 under spinal, 6 under general). After inspection of the perianal skin and identification of the external fistula opening(s), the anal canal was examined using an anal retractor. The internal opening was identified either by gentle probing with a metal probe or by instillation of hydrogen peroxide through the external opening. If the internal opening could not be identified, forceful probing was avoided to prevent the creation of false tracts or openings.

**FIGURE 1:**
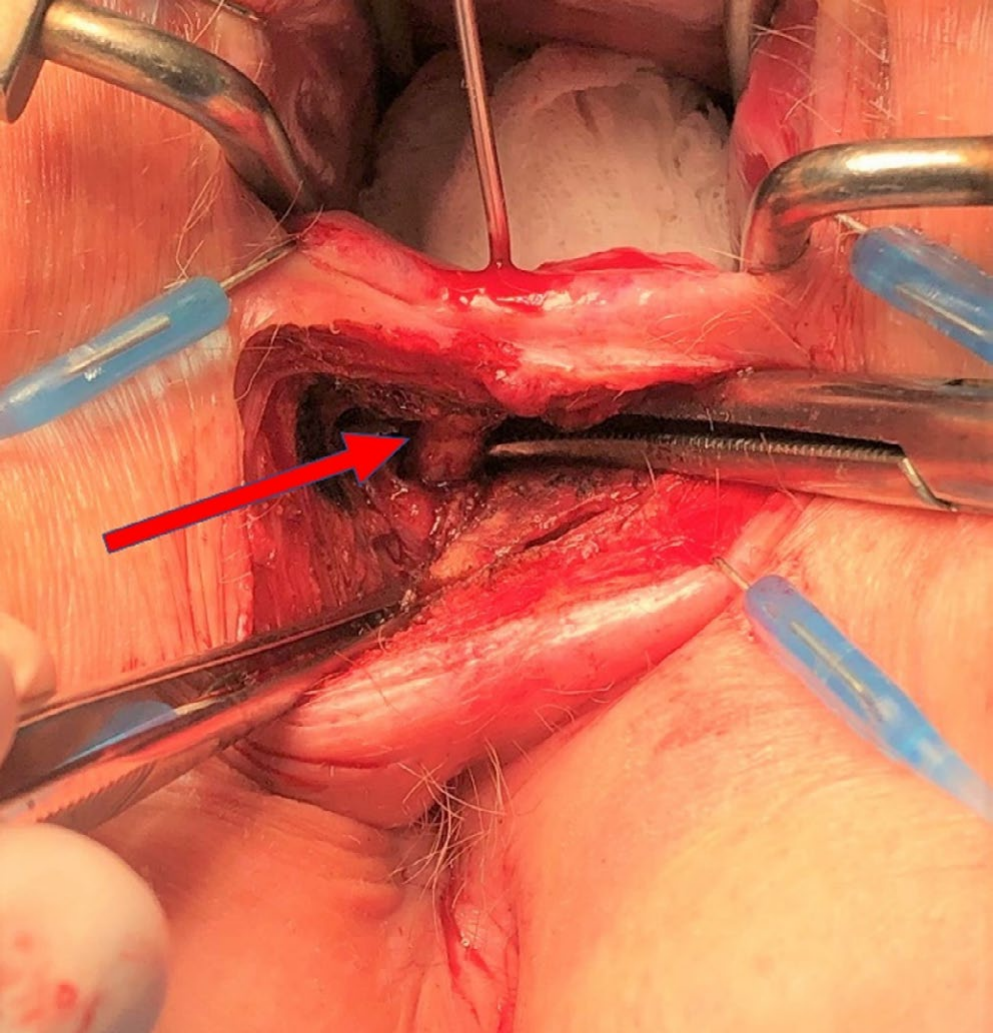
Preparation of the intersphincteric portion of the fistula tract, red arrow showing fistula tract.

A curvilinear incision, 2–3 centimetres in length, is made at the intersphincteric groove using electrocautery, followed by dissection of the intersphincteric space up to the intersphincteric portion of the anal fistula tract (Figure 1).

After the intersphincteric portion of the fistula tract is identified and dissected, it is excised along with the presumed anal gland, which is considered the initial source of the pathology. At this stage, additional curettage of all granulation tissue and debris from the fistula tract is performed, followed by irrigation with a saline solution.

In the next step, we close the remaining defect in the internal anal sphincter muscle through the intersphincteric space using an absorbable 3‐0 suture, after which the defect in the external sphincter is addressed in the same fashion. This step is similar to that in the LIFT procedure.

To ensure adequate postoperative drainage, the external fistula opening is enlarged by excising the surrounding tissue. A portion of nearby skin, unaffected by inflammation, is used as a free graft for implantation in the intersphincteric space – an essential step of the ASGIIFT technique. Excision is performed sharply with a scalpel to avoid thermal damage. A 1 × 1 cm skin graft, 3–4 mm thick, is prepared by removing the epidermis and fat, leaving only the dermis. The graft is rinsed with saline and handled carefully to prevent damage.

**FIGURE 2:**
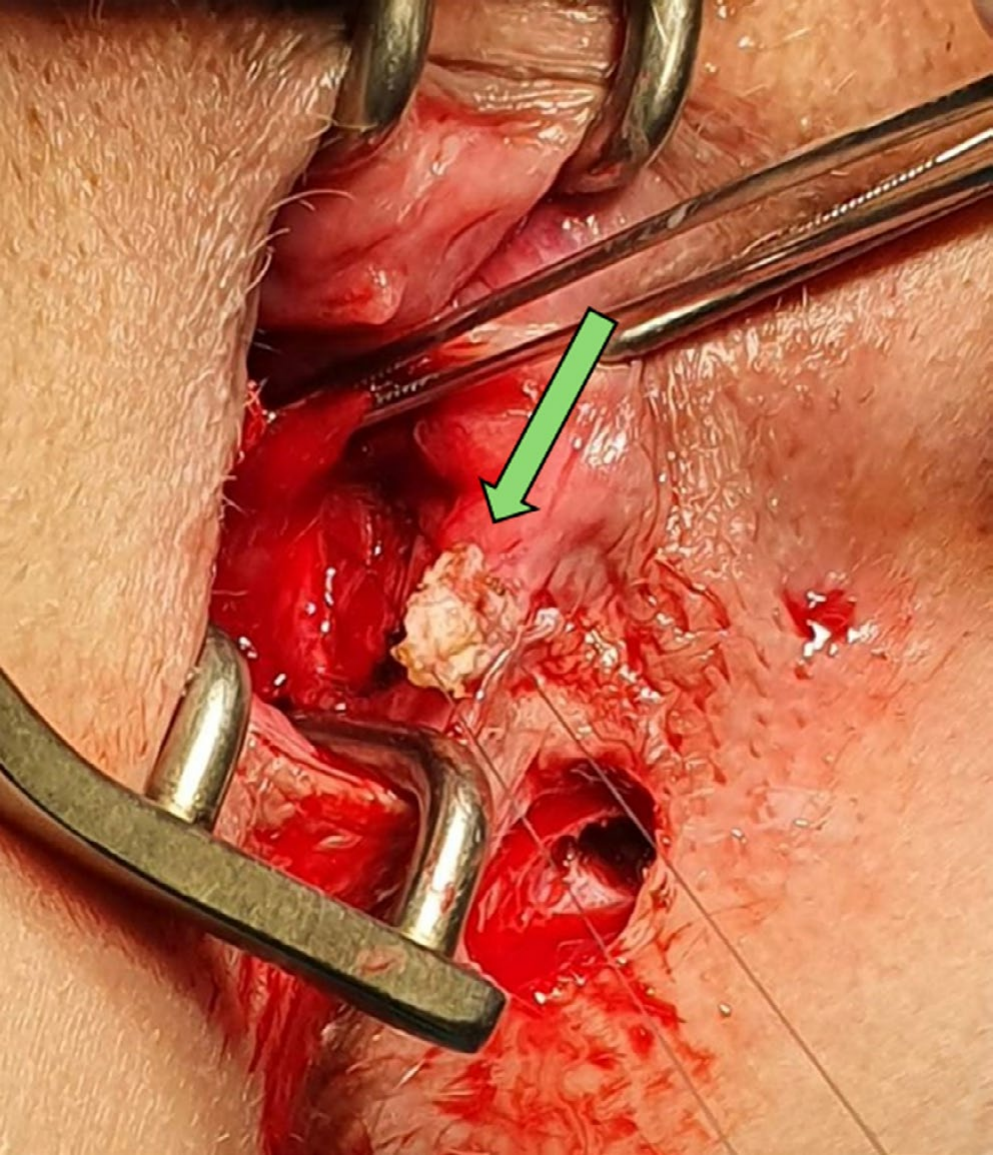
Autologous skin graft prepared for implantation.

**FIGURE 3:**
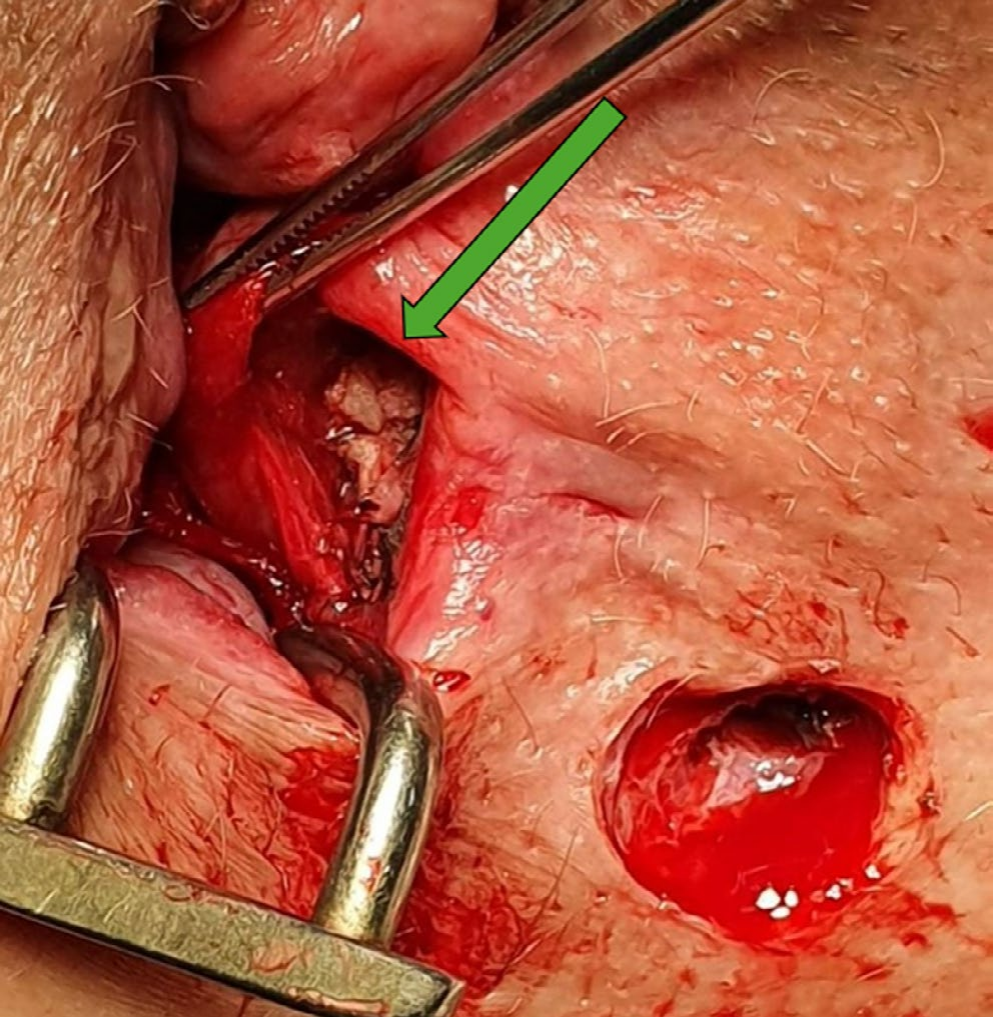
Autologous skin graft fixed in intersphincteric space.

The autologous graft is implanted in the intersphincteric space using an absorbable 4‐0 suture, anchored to the internal anal sphincter at the site of muscle repair. It is then lowered into place, the knots tied and the edges secured with interrupted sutures, functioning like a “sticky gum” to reinforce the closure (Figures 2 and 3). Closure of the intersphincteric space is then performed using interrupted absorbable 3‐0 sutures.

#### Postoperative management

All patients were discharged on the day of surgery (day‐case procedure) or the following day, with a recommendation to continue oral antibiotics for 7 days (ciprofloxacin 500 mg twice daily and metronidazole 400 mg three times daily). Patients were instructed to shower the wound 2–3 times daily – especially after each bowel movement – and to apply appropriate wound dressings. A fibre‐rich diet was recommended, along with cessation of smoking and alcohol.

#### Outcomes and data analysis

The primary outcome was the postoperative primary healing rate, which was defined clinically. Secondary outcomes included postoperative continence disturbance, postoperative pain, time to healing and other postoperative complications (assessed using the Wexner score and Visual Analogue Scale – VAS), as well as comparison of the initial results of the ASGIIFT technique with published outcomes of other similar sphincter‐preserving techniques, such as the LIFT procedure.

We used Microsoft Excel (Microsoft, Seattle, WA) spreadsheet for data collection. Descriptive statistics were used to summarise the data; no inferential tests were performed due to the limited sample size and single‐arm design.

## RESULTS

Forty (40) adult patients over 18 years of age (29 men, 11 women; mean age 41.8 years) with either primary (*n* = 32) or recurrent (*n* = 8) complex cryptoglandular transsphincteric anal fistulas were included in this study. None of the patients had serious preoperative continence disturbances (in two patients, the Wexner score was 2).

Of the 8 patients with recurrent fistulas (who had operations for anal fistulas prior to the ASGIIFT procedure), 6 had previously undergone the VAAFT (video‐assisted anal fistula treatment) procedure and 2 had undergone the LIFT procedure, at least 4 months prior to the ASGIIFT procedure (range 4–18 months).

Among the 40 included patients, 12 patients (30%) had previously received a loose seton for at least 4 months: 6 with recurrent fistulas who had operations prior to the ASGIIFT procedure (4 after VAAFT and 2 after LIFT) and 6 who were initially treated by incision of a perianal abscess during which the seton was placed. Of these patients with seton, 10 (83.3%) achieved successful healing postoperatively.

The internal fistula opening was identified in 34 cases (85%). The internal opening was verified in all patients who had previous operations before the ASGIIFT procedure (8 cases) and in 26 cases in patients for whom the ASGIIFT procedure was the first operation for anal fistula treatment.

From the 34 cases in which the internal fistula opening was identified, 33 initially healed after the ASGIIFT procedure (1 did not succeed), but in long‐term follow‐up, 2 additional patients experienced recurrence. In the group without identification of the internal fistula opening (total 6 cases), 2 patients had recurrence during the follow‐up period.

Primary healing was initially achieved in 37 patients within a median of 4 weeks postoperatively (range: 3–6 weeks). However, two additional patients experienced recurrence during follow‐up – one after 3 months and the other after 4 months – indicating that sustained healing was ultimately achieved in 35 out of 40 patients (87.5%).

Fifteen (15) patients were smokers (37.5%).

Among the 5 patients who did not achieve primary healing, 3 were non‐smokers and 2 were smokers. Additionally, 3 of the non‐healed cases were men and 2 were women. Eight patients (20%) had recurrent fistulas at baseline (had previous operations prior to the ASGIIFT procedure). Following the ASGIIFT procedure in these 8 patients, healing was achieved in 7 cases (87.5%).

Most patients reported only mild postoperative pain. Pain was assessed using the Visual Analogue Scale (VAS), with a mean score of 2.5 (range: 0–5) on a scale from 0 to 10 during the first 48 h.

None of the patients reported any postoperative continence disturbances. The only two patients who had preoperative continence issues (Wexner score of 2) did not experience postoperative worsening of continence.

The median duration of the operation was 35 min (range: 20–45 min). The median time to return to daily activities was 25 days (range: 14–45 days) (Table 1).

**TABLE 1:** Clinical outcomes according to patient characteristics.

Variable	*n*	Initial healing, *n* (%)	Recurrence during follow‐up, *n* (%)	Sustained healing, *n* (%)
Total	40	37 (92.5)	2 (5.0)	35 (87.5)
Sex				
Male	29	26 (89.7)	2 (6.9)	24 (82.8)
Female	11	11 (100)	0	11 (100)
Smoking status				
Smoker	15	13 (86.7)	1 (6.7)	12 (80.0)
Non‐smoker	25	24 (96.0)	1 (4.0)	23 (92.0)
Fistula type				
Primary	32	30 (93.8)	2 (6.3)	28 (87.5)
Recurrent (had prior operations)	8	7 (87.5)	0	7 (87.5)
Preoperative seton				
Yes	12	10 (83.3)	0	10 (83.3)
No	28	27 (96.4)	2 (7.1)	25 (89.3)
Internal opening identified				
Yes	34	33 (97.1)	2 (5.9)	31 (91.2)
No	6	4 (66.7)	2 (33.3)	2 (33.3)
Minor wound dehiscence	3	0	3 (100)	0
Conversion to intersphincteric fistula	5	0	0	5 (100)

All 40 patients included in the study completed the 18‐month follow‐up.

None of the patients experienced serious complications. However, two patients developed postoperative urinary retention and three patients had minor wound dehiscence at the site of the intersphincteric approach during follow‐up – all of whom failed to achieve postoperative fistula healing (they had diversion from transsphincteric to intersphincteric fistula).

Five initially unhealed patients (all had posterior fistulas) showed conversion from transsphincteric to intersphincteric fistula during the follow‐up period and were subsequently treated by fistulotomy without complications.

No obvious trends were observed regarding primary healing according to smoking status, gender, or prior seton placement.

## DISCUSSION AND CONCLUSION

The primary outcome of this study was the postoperative healing rate after the ASGIIFT procedure. In our study, ASGIIFT achieved primary healing in 87.5% of patients within a median of four weeks, without deterioration in continence or major complications. Among the five patients who did not achieve initial healing, all converted from transsphincteric to simple intersphincteric fistulas and were successfully treated with additional fistulotomy, without the development of incontinence. Thus, all 40 patients included in the study ultimately achieved complete fistula healing, with no significant complications. These findings confirm that the technique is feasible, safe and associated with rapid recovery, fulfilling the goals of this IDEAL stage 2a evaluation.

Compared with published results of other sphincter‐preserving procedures, such as the LIFT and BioLIFT techniques, the outcomes of ASGIIFT are encouraging. Although Rojanasakul initially reported a high success rate of 94.4%, subsequent studies showed variable results. For instance, Hong et al. [19, 20] in a systematic review and meta‐analysis of 24 original articles (including 1110 patients, average follow‐up of 10.3 months), reported a mean success rate of 76.4%, with incontinence, intraoperative and postoperative complication rates of 0%, 0% and 5.5%, respectively. Ellis introduced the BioLIFT procedure, involving placement of a bioprosthetic graft in the intersphincteric space. In his study of 31 patients, 29 (94%) achieved clinical healing, with no complications requiring intervention [17].

By using autologous dermal tissue, ASGIIFT provides a biologically active and inexpensive graft that may promote angiogenesis and healing while avoiding prosthetic‐related complications. These principles, recognised in reconstructive surgery, may explain the favourable healing observed in this series [18].

Secondary outcomes further support the safety and functional preservation of this technique. No new continence disturbances occurred, postoperative pain was mild, and complications were minimal, similar to those of previously published techniques [17, 19, 20]. These findings align with previous studies emphasising that preservation of the sphincter complex is critical for maintaining postoperative function [2, 3, 4, 5, 6, 7, 8, 9, 10, 11, 12, 13, 14, 15, 16, 17]. Notably, the average healing time observed in this study was shorter than in our previously published studies, decreasing from 6 to 4 weeks [21].

As mentioned in the Results section, only 12 patients had a preoperatively placed loose seton: 6 of these had previously undergone surgery (4 with the VAAFT technique and 2 with the LIFT technique), while the remaining 6 had a seton placed following incision of a perianal abscess once the internal fistula opening was identified. We do not routinely place a seton prior to the LIFT procedure, except in cases of intersphincteric collections (abscesses) or larger sphincter defects resulting from chronic inflammation secondary to the fistula. In such cases, as well as in patients with complex fistulas extending deep into the ischioanal fossa, the fistula is often initially treated with the VAAFT procedure and a loose seton placement, followed by definitive sphincter‐sparing surgery after a minimum interval of 3–4 months. This approach is consistent with the findings of Placer‐Galán et al. [22], who reported no significant differences in recurrence rates between patients with or without pre‐LIFT seton placement.

Factors potentially influencing failure of the ASGIIFT technique include deviations from the procedural protocol. Strict adherence is essential at all stages: preoperative measures (bowel preparation, antibiotic prophylaxis), intraoperative steps (careful technique, minimal electrocautery, avoiding graft trauma, irrigating only with saline) and postoperative management (smoking and alcohol avoidance, close outpatient follow‐up with regular wound care). Future studies should prospectively evaluate these factors using multivariate analysis to identify predictors of healing and recurrence, ideally in larger, multicentre cohorts.

### Limitations and conclusion

This study has several limitations that should be considered when interpreting the results. Given that this is an IDEAL 2a stage study, its primary aim was to evaluate feasibility and safety in a limited patient series, which inherently restricts the generalisability of the findings. It is a single‐centre, single‐arm prospective observational study with a relatively small sample size. The absence of a control group prevents direct comparison with other sphincter‐preserving techniques, such as LIFT or BioLIFT, in terms of healing rates, recurrence, or functional outcomes. Follow‐up relied primarily on clinical evaluation without routine imaging, which may have missed subclinical recurrences. Although all procedures were performed by experienced coloproctologists using a standardised protocol, operator‐dependent variability may still have influenced outcomes. Finally, patient‐related factors, such as smoking, prior surgeries and complexity of fistula tracts, may have affected healing, and their influence could not be fully analysed due to the limited sample size.

Future studies should address these limitations through multicentre, randomised controlled trials with larger patient populations, standardised imaging follow‐up and formal analysis of potential predictive factors to validate the safety and efficacy of the ASGIIFT procedure.

## AUTHOR CONTRIBUTIONS

**Damir Karlović:** Conceptualization; methodology; investigation; data curation; writing – original draft; supervision; validation. **Dorian Kršul:** Investigation; data curation; validation. **Dora Fučkar Čupić:** Formal analysis; writing – review and editing; validation. **Marko Zelić:** Formal analysis; writing – review and editing; validation. All authors: Review and editing, approval of final manuscript.

## FUNDING INFORMATION

The authors have nothing to report.

## CONFLICT OF INTEREST STATEMENT

The authors have no conflicts of interest relevant to the content of this manuscript.

## ETHICS STATEMENT

The patients provided written informed consent for the procedure, and the study was approved by the Ethics Committee of University Hospital Rijeka.

## PATIENT CONSENT STATEMENT

Informed consent was obtained from all individual participants included in the study.

## Data Availability

The data that support the findings of this study are available on request from the corresponding author. The data are not publicly available due to privacy or ethical restrictions.

## References

[1] Vogel JD , Johnson EK , Morris AM , Paquette IM , Saclarides TJ , Feingold DL , et al. Clinical practice guideline for the management of anorectal abscess, fistula‐in‐ano, and rectovaginal fistula. Dis Colon Rectum. 2016;59(12):1117–1133. 27824697 10.1097/DCR.0000000000000733

[2] Atkin GK , Martins J , Tozer P , Ranchod P , Phillips RKS . For many high anal fistulas, lay open is still a good option. Tech Coloproctol. 2011;15(2):143–150. 21431388 10.1007/s10151-011-0676-6

[3] Ritchie RD , Sackier JM , Hodde JP . Incontinence rates after cutting seton treatment for anal fistula. Color Dis. 2009;11(6):564–571. 10.1111/j.1463-1318.2008.01713.x 19175623

[4] van Koperen PJ , Wind J , Bemelman WA , Bakx R , Reitsma JB , Slors JFM . Long‐term functional outcome and risk factors for recurrence after surgical treatment for low and high perianal fistulas of cryptoglandular origin. Dis Colon Rectum. 2008;51(10):1475–1481. 18626715 10.1007/s10350-008-9354-9

[5] Garg P . A new understanding of the principles in the management of complex anal fistula. Med Hypotheses. 2019;132:109329. 31421428 10.1016/j.mehy.2019.109329

[6] Meinero P , Mori L . Video‐assisted anal fistula treatment (VAAFT): a novel sphincter‐saving procedure for treating complex anal fistulas. Tech Coloproctol. 2011;15(4):417–422. 22002535 10.1007/s10151-011-0769-2 PMC3226694

[7] Rojanasakul A . LIFT procedure: a simplified technique for fistula‐in‐ano. Tech Coloproctol. 2009;13(3):237–240. 19636496 10.1007/s10151-009-0522-2

[8] Balciscueta Z , Uribe N , Balciscueta I , Andreu‐Ballester JC , Garcia‐Granero E . Rectal advancement flap for the treatment of complex cryptoglandular anal fistulas: a systematic review and meta‐analysis. Int J Color Dis. 2017;32:599–609. 10.1007/s00384-017-2779-7 28247060

[9] Giamundo P , Esercizio L , Geraci M , Tibaldi L , Valente M . Fistula‐tract laser closure (FiLaC™): long‐term results and new operative strategies. Tech Coloproctol. 2015;19(8):449–453. 25724967 10.1007/s10151-015-1282-9

[10] Verkade C , Zimmerman DDE , Wasowicz DK , Polle SW , de Vries HS . Loss of seton in patients with complex anal fistula: a retrospective comparison of conventional knotted loose seton and knot‐free seton. Tech Coloproctol. 2020;24(10):1043–1046. 32562152 10.1007/s10151-020-02254-1

[11] Litta F , Parello A , De Simone V , Grossi U , Orefice R , Ratto C . Fistulotomy and primary sphincteroplasty for anal fistula: long‐term data on continence and patient satisfaction. Tech Coloproctol. 2019;23(10):993–1001. 31538298 10.1007/s10151-019-02093-9

[12] Beaulieu R , Bonekamp D , Sandone C , Gearhart S . Fistula‐in‐ano: when to cut, tie, plug, or sew. J Gastrointest Surg. 2013;17(6):1143–1152. 23315048 10.1007/s11605-012-2126-9

[13] Mennigen R , Laukötter M , Senninger N , Rijcken E . The OTSC® proctology clip system for the closure of refractory anal fistulas. Tech Coloproctol. 2015;19:241–246. 25715788 10.1007/s10151-015-1284-7

[14] Seow‐Choen F , Nicholls RJ . Anal fistula. Br J Surg. 1992;79:197–205. 1555083 10.1002/bjs.1800790304

[15] Parks AG , Gordon PH , Hardcastle JD . A classification of fistula‐in‐ano. Br J Surg. 1976;63(1):1–12. 1267867 10.1002/bjs.1800630102

[16] Matos D , Lunniss PJ , Phillips RKS . Total sphincter conservation in high fistula in ano: results of a new approach. Br J Surg. 1993;80:802–804. 8330181 10.1002/bjs.1800800651

[17] Ellis CN . Outcomes with the use of bioprosthetic grafts to reinforce the ligation of the intersphincteric fistula tract (BioLIFT procedure) for the management of complex anal fistulas. Dis Colon Rectum. 2010;53(10):1361–1364. 20847616 10.1007/DCR.0b013e3181ec4470

[18] Kohlhauser M , Luze H , Nischwitz SP , Kamolz LP . Historical evolution of skin grafting – a journey through time. Medicina (Kaunas). 2021;57(4):348. 33916337 10.3390/medicina57040348 PMC8066645

[19] Rojanasakul A , Pattana‐arun J , Sahakitrungruang C , Tantiphlachiva K . Total anal sphincter saving technique for fistula‐in‐ano: the ligation of intersphincteric fistula tract. J Med Assoc Thai. 2007;90:581–586. 17427539

[20] Hong KD , Kang S , Kalaskar S , Wexner SD . Ligation of intersphincteric fistula tract (LIFT) to treat anal fistula: systematic review and meta‐analysis. Tech Coloproctol. 2014;18(8):685–691. 24957361 10.1007/s10151-014-1183-3

[21] Zelić M , Karlović D , Kršul D , Bačić Đ , Warusavitarne J . Video‐assisted anal fistula treatment for treatment of complex cryptoglandular anal fistulas with 2 years follow‐up period: our experience. J Laparoendosc Adv Surg Tech A. 2020;30(12):1329–1333. 32412822 10.1089/lap.2020.0231

[22] Placer‐Galan C , Enriquez‐Navascues JM , Pastor‐Bonel T , Aquirre‐Allende I , Saralegui‐Ansorena Y . The use of seton as a bridge to definitive ligation of the intersphincteric fistula tract procedure for fistula‐in‐ano: a systematic review and meta‐analysis. J Coloproctol. 2021;41(3):308–1333. doi:10.1055/s-00411730039

